# Hepatitis E Virus: What More Do We Need to Know?

**DOI:** 10.3390/medicina60060998

**Published:** 2024-06-18

**Authors:** Endrit Shahini, Antonella Argentiero, Alessandro Andriano, Francesco Losito, Marcello Maida, Antonio Facciorusso, Raffaele Cozzolongo, Erica Villa

**Affiliations:** 1 Gastroenterology Unit, National Institute of Gastroenterology-IRCCS “Saverio de Bellis”, Castellana Grotte, 70013 Bari, Italy; francesco.losito@irccsdebellis.it (F.L.); raffaele.cozzolongo@irccsdebellis.it (R.C.); 2 Istituto Tumori “Giovanni Paolo II”, Medical Oncology Unit, 70124 Bari, Italy; argentieroantonella@gmail.com; 3 Department of Precision and Regenerative Medicine and Ionian Area, University of Bari Aldo Moro Medical School, 70124 Bari, Italy; a.andriano2@studenti.uniba.it; 4 Gastroenterology and Endoscopy Unit, S. Elia-Raimondi Hospital, 93100 Caltanissetta, Italy; marcello.maida@hotmail.it; 5 Department of Medical and Surgical Sciences, University of Foggia, 71122 Foggia, Italy; antonio.facciorusso@unifg.it; 6 Gastroenterology Unit, CHIMOMO Department, University of Modena & Reggio Emilia, Via del Pozzo 71, 41121 Modena, Italy

**Keywords:** HEV, viral cell entry, inhibitors, antiviral treatment, pregnancy, chronic infection

## Abstract

Hepatitis E virus (HEV) infection is typically a self-limiting, acute illness that spreads through the gastrointestinal tract but replicates in the liver. However, chronic infections are possible in immunocompromised individuals. The HEV virion has two shapes: exosome-like membrane-associated quasi-enveloped virions (eHEV) found in circulating blood or in the supernatant of infected cell cultures and non-enveloped virions (“naked”) found in infected hosts’ feces and bile to mediate inter-host transmission. Although HEV is mainly spread via enteric routes, it is unclear how it penetrates the gut wall to reach the portal bloodstream. Both virion types are infectious, but they infect cells in different ways. To develop personalized treatment/prevention strategies and reduce HEV impact on public health, it is necessary to decipher the entry mechanism for both virion types using robust cell culture and animal models. The contemporary knowledge of the cell entry mechanism for these two HEV virions as possible therapeutic target candidates is summarized in this narrative review.

## 1. Introduction

Hepatitis E virus (HEV), an established human pathogen, infects humans and various other animal species [1]. HEV spreads directly by contact with infected animals, contaminated food, or the environment [1].

In 2016, HEV was classified as a “quasi-enveloped” virus (eHEV), with both non-enveloped (“naked”) and enveloped forms, similar to HAV [1]. HEV is shed in feces as a non-enveloped virus, but in cell culture, it has a lipid envelope [1].

HEV is a poorly understood zoonotic virus that causes acute and chronic viral hepatitis [2,3]. The incidence of chronic HEV infection/chronic hepatitis is extremely low, owing primarily to immune-deficient status (i.e., transplant recipients, human immunodeficiency virus patients with low CD4 counts, and patients with hematologic malignancies receiving chemotherapy) [3].

HEV infection is widespread worldwide, with distinct differences in transmission and disease outcomes in resource-rich versus resource-limited areas. It is estimated that 20 million HEV infections occur annually, resulting in 3.4 million clinical cases of hepatitis E and 70,000 hepatitis E-related deaths worldwide [4].

Disease manifestation, infection source, and route of transmission vary by HEV genotype and epidemiology in both endemic and non-endemic global settings [1,2,3,4]. In developed countries, hepatitis E occurs primarily irregularly or in clusters, whereas in developing countries, endemic or epidemic hepatitis E is occasionally associated with large outbreaks [5]. Specifically, minority populations with serious social problems, such as poverty, low income, unemployment, a lack of education, and inadequate housing, are more likely to contract HEV infection [6].

The primary route of HEV transmission is fecal–oral via contaminated drinking water or consumption of raw animal meat products [7]. HEV infects many species, including bats, ferrets, rabbits, and chickens. However, swine, deer, and wild boar are the primary reservoirs for transmission to humans [1].

In 1978, HEV was identified as an epidemic of non-A and non-B hepatitis from Kashmir, India [8]. Then, since the discovery of swine HEV in pigs in the United States (1997) [9], novel strains of HEV have been genetically identified from over a dozen animal species, including domestic and rabbit, wild pig, rat, camel, mongoose, and chick [5,6,9,10,11,12].

This review will elucidate the mechanism of viral cell entry and current and alternative therapies for HEV infection. Antivirals inhibit the viral life cycle and have distinct advantages in suppressing viral infections, making them of particular interest due to their potential therapeutic or prophylactic applications. This premise is especially critical for immunosuppressed chronic HEV infection subjects who have failed conventional therapy and pregnant women with HEV-induced severe hepatic injury, even leading to lethal cases and fetus death.

## 2. HEV Genotypes

Several HEV genotypes have varying degrees of host specificity [13]. There have been eight HEV genotypes identified so far [14].

HEV genotypes 1 and 2 infect only humans and primates, whereas genotypes 3 and 4 infect both humans and other mammals (including deer, swine, dolphins, cows, nonhuman primates, and bears) [15,16]. Genotype 3 is most commonly found in industrialized countries (Europe and America) [15]. Furthermore, HEV genotype 3 infection is usually asymptomatic, but it can induce severe acute hepatitis in patients with chronic liver disease, as well as chronic hepatitis and cirrhosis in immunocompromised patients [15]. HEV genotypes 5 and 6 have been isolated from wild boars, while genotypes 7 and 8 have been identified in camels from the United Arab Emirates and China, respectively [14].

Interestingly, rodent-associated HEV is gaining particular attention because it can infect humans as a secondary host and cause severe acute and/or chronic hepatitis [16]. Additionally, HEV genotypes have specific differences in prevalence by geographic region.

## 3. Virology

### 3.1. HEV Composition and Structure

HEV is a small, icosahedral, non-enveloped 7.2 kb single-stranded positive-sense RNA virus that belongs to Paslahepivirus balayani according to the current nomenclature (similar to hepatitis A virus of the Picornaviridae) with a 27–34 nm diameter [17,18,19,20,21].

There are currently eight known HEV genotypes [20]. The majority of the strains that infect humans (HEV genotypes from 1 to 4 and genotype 7) belong to two species: orthohepevirus A (HEV genotypes from 1 to 8) and orthohepevirus C [15].

Virions shed in feces are non-enveloped, whereas virions are released in cell culture and circulating in the blood as quasi-enveloped due to a lipid envelope [22].

HEV causes self-limiting acute hepatitis in humans [23]. Capsomeres are composed of a single capsid protein homodimer forming the HEV capsid. Each capsid protein contains three domains (with a polysaccharide-binding site that may interact with cellular receptors) that form distinct structural elements: S (constant capsid), P1 (three-fold protrusions), and P2 (two-fold spikes with neutralizing epitopes) [23].

The HEV genome organization is similar to many alphaviruses, with a methyl guanosine cap (m7G) and nonstructural genes at the 5′ end and structural genes at the 3′ end with polyadenylated (PolyA) tail [23]. The 5′ untranslated region of HEV is 28 nucleotides long, and bases 2–26 have the potential to form a complex secondary structure or hairpin [23].

**Figure 1:**
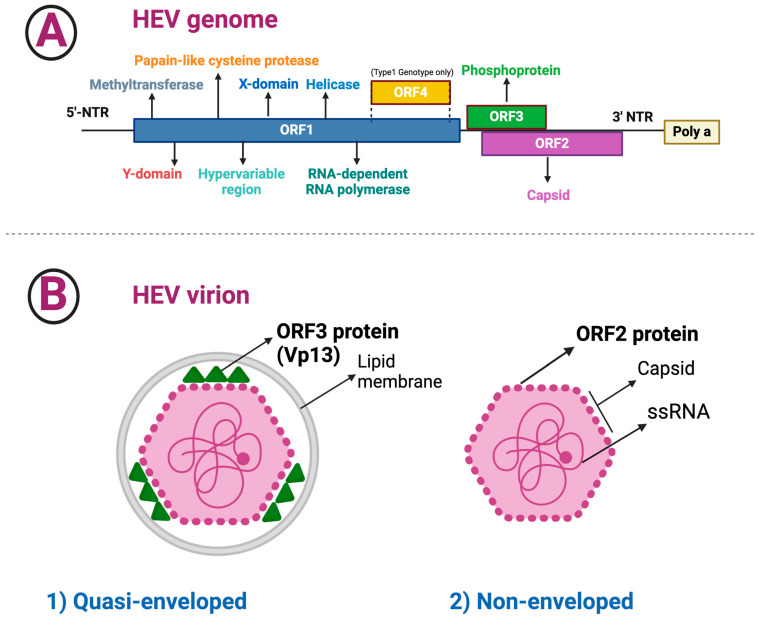
Hepatitis E virus (HEV) genome and virion structure. The figure illustrates the genomic organization and virion structure of the Hepatitis E virus (HEV). (**A**) Genomic Organization: The HEV genome is a 7.2 kilobase positive-sense single-stranded RNA molecule. It is organized with nonstructural genes at the 5′ end and a methyl guanosine cap (m7G), as well as structural genes at the 3′ end with polyadenylated (PolyA) tail. The 5′ untranslated region of HEV is 28 nucleotides long, with bases 2–26 having the potential to form a complex secondary structure. Furthermore, the HEV genome is polyadenylated at the 3′ end and consists of three partially overlapping open reading frames (ORFs). It is depicted as a linear arrangement of genetic elements, including these three ORFs. ORF1 produces nonstructural proteins that are required for viral replication. ORF2 produces the viral capsids, which form the viral particle’s capsid. ORF3 is a small multifunctional protein, wrapped by a membrane, which is necessary for virion release and pathogenesis. (**B**) Virion organization: The HEV virus has two varieties: (1) quasi-enveloped virions, which have an outer membrane analogous to exosomes and have been isolated from HEV-infected cells’ bloodstream and cell culture supernatant; (2) non-enveloped virions found in the feces and bile of infected hosts.

The genome of HEV is polyadenylated at the 3′ end and consists of three partly overlapping open reading frames (ORFs) (Figure 1) [24]:

▪ The length of ORF1 varies among HEV genotypes; ORF1 codes for nonstructural proteins involved in virus replication (i.e., methyltransferase, Y domain, papain-like cysteine protease, hypervariable region, X or macro domain, helicase, and RNA-dependent RNA polymerase) [25].
▪ The ORF2 has 112–114 codons that are divided into three structural domains [shell (S), middle (M), and protruding (P)] [20,26]. HEV encodes two isoforms of the ORF2 capsid protein, which are distributed in different subcellular compartments and serve distinct functions throughout the HEV lifecycle [27]. Recombinant ORF2 proteins expressed in bacterial and insect cells have been shown to self-assemble into virus-like particles, the crystal structures of which have been determined [20,21,28,29]. The ORF2 protein was found to bind specifically to the 5′ end of the HEV genome [23]. Several techniques for amplifying the ORF2 gene have been reported, as the ORF2 gene is frequently utilized to analyze and genotype HEV strains [30].
▪ ORF3 has 123 codons and encodes a small phosphoprotein, a functional iron channel that appears to function as a viroporin, allowing infectious virions to be released from infected cells [31].
▪ ORF4, a new ORF discovered within the coding region of ORF1, appears to be restricted to genotype 1 HEV and is translated into a protein that increases activity of the RNA-dependent RNA polymerase [1,18,32,33]; p-ORF4 consists of 124 aa, promotes replication of the virus, is indispensable for the life cycle of HEV genotype 1, and is possibly involved in the increased severity of infection in pregnancy [1,18].

The HEV virion comes in two types: eHEV, which are covered by an external membrane similar to exosomes and have been isolated from the bloodstream and cell culture supernatant of HEV-infected cells, and non-enveloped virions, which were found in infected hosts’ feces and bile [34,35,36]. The simultaneous presence of the quasi-enveloped and non-enveloped viruses reflects HEV’s versatility in various host environments. The lipid envelope of quasi-enveloped virions may improve bloodstream survival and cell entry, whereas the stability of non-enveloped virions in feces and bile environments promotes efficient transmission via the fecal–oral route.

In isopycnic gradient centrifugation, eHEV particles band at a much lower buoyant density (~1.10 g/cm^3^) than traditional, non-enveloped virions (~1.25 g/cm^3^) [34] and are insensitive to neutralizing anti-capsid antibodies in standard neutralization assays [37].

### 3.2. The Viral Cell Entry, Endosomal Transport and Uncoating

Although HEV is primarily transmitted enterically, it is unclear how it crosses the gut barrier and enters the portal bloodstream, and the first round of infection requires quasi-enveloped HEV [33] or infrequently non-enveloped HEV [38].

HEV gut infection was demonstrated in a pig model, where the HEV RNA was detected by PCR in small intestinal and colonic tissue. HEV was found to replicate efficiently in polarized Caco-2 human colonic adenocarcinoma cells [38].

The inadequacy of successful HEV cell cultivation techniques hindered HEV life cycle research [38]. In studies conducted in heterologous settings, cellular factors interacting with HEV proteins that need to be validated and studied further have been identified [39,40].

The contemporary growth of HEV infection methods has allowed for the thorough investigation of the whole life cycle of HEV for the first time [41,42]. In vitro, HEV has a broad and distinct cell tropism. Marion et al. [43] developed various cell culture systems (exploiting tissue derived from small bowel resections) using as infecting agents HEV genotype 1 and 3 isolates from chronic hepatitis E (CHE) patients’ stool samples, as well as virus derived from the genotype 3 Kernow-C1 p6 clone. They showed that HEV can infect intestinal cells and provided the first evidence for productive HEV infection of authentic human primary intestinal cells.

HEV can cross the intestinal vascular endothelium and travel to the liver via the portal venous system, infecting hepatocytes from the basolateral side [38]. HEV replicates in intestinal cells and releases infectious particles from both the basolateral membrane and (preferentially) apical sides of (polarized) primary enterocytes before entering the bloodstream and liver as quasi-enveloped virions (low density), resulting in viremia of membrane-cloaked infectious virions [33,38]. Furthermore, non-enveloped HEV virions without a “quasi-envelope” can be found in the blood of some infected subjects (less than 20% of the circulating virus), especially late in infection when the liver is damaged [33].

**Figure 2:**
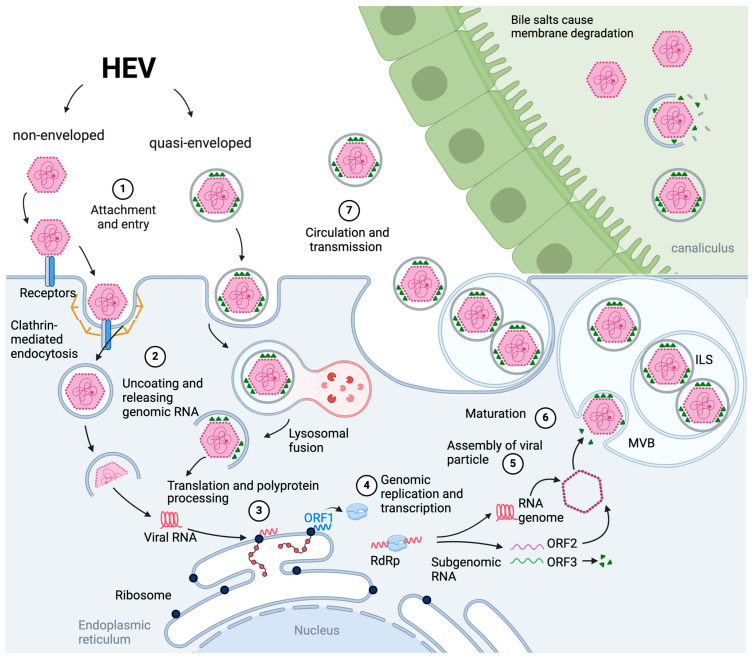
Hepatitis E virus (HEV) viral life cycle. 1. Attachment and Entry: Non-enveloped HEV attaches to specific receptors on the surface of susceptible host cells, including hepatocytes in the liver. This interaction is mediated by clathrin, heparan sulfate, integrin alpha 3, and dynamin 2 molecules. This engagement triggers receptor-mediated endocytosis, leading to viral host cell internalization. Alternatively, evidence shows that quasi-enveloped HEV enters cells via endocytosis followed by degradation of the membrane within the endolysosome. 2. Uncoating and Releasing of Genomic RNA: Once inside the host cell, HEV’s viral capsid undergoes disassembly within the endosome. The precise mechanism of HEV capsid uncoating remains still unclear. 3. Translation and Polyprotein Processing: Within the cytoplasm, the HEV genomic RNA is translated into a large polyprotein. The nonstructural proteins encoded by ORF1 play pivotal roles in viral replication and other processes. 4. Genome Replication and Transcription: The HEV RNA-dependent RNA polymerase (RdRp), a product of ORF1, catalyzes the HEV genome replication and transcription. This step results in the production of novel viral RNA genomes and subgenomic RNA species. 5. Assembly of Viral Particles: The newly synthesized viral genomic RNA is encapsidated by the structural capsid protein encoded by ORF2. This encapsidation process forms complete viral particles within the cytoplasm. 6. Maturation and Transport: The mature viral particles undergo structural changes and assemble into quasi-enveloped, spherical virions. The process of viral release from the host cell likely involves the endosomal sorting complexes required for transport (ESCTR) machinery. 7. Circulation and Transmission: After the fusion of multivesicular bodies (MVB) with plasma membranes, viral particles are released via the exosomal pathway so that only quasi-enveloped enters the bloodstream. Canalicular bile salts cause the degradation of the coating membrane and the production of naked particles. These particles reach the environment through bile secretion into the intestine, becoming the major source of contamination and transmission.

The virus’s entry process may vary since HEV exists in both non-enveloped (“naked”) and quasi-enveloped HEV shapes (Figure 2). The discovery of quasi-enveloped HEV particles completely changed the understanding of the HEV life cycle and pathogenesis, as evidenced by recent research that revealed that quasi-enveloped HEV particles are transmissible and are most likely uniquely responsible for infection within the host. The capsid would have to cross two layers of membranes to deliver the HEV genome into the cytoplasm in the presence of an eHEV.

Figure 2 depicts a comprehensive overview of the HEV viral life cycle.

Although some HEV entry elements and mechanisms were determined, key variables remain unidentified. HEV’s infectious capacity of a wide range of cells [41,42,43,44,45,46,47,48], which may be related to the various extrahepatic manifestations of hepatitis E [49,50,51,52], strongly suggests that unknown entry factors may be widely expressed. However, several host factors have been implicated in cell adherence and/or entry of naked HEV. Phosphatidylserine is found in the eHEV membrane and can attach to its receptor on target cells, such as TIM-1 [53,54]. Due to inadequate cell attachment, cellular intake of eHEV virions is less effective than that of naked HEV [34]. Studies using virus-like particles (VLP) as a model system have identified potential host factors involved in virus attachment and internalization, such as the 78 kDa glucose-regulated protein (GRP78), ATP synthase subunit (ATPB5), and asialoglycoprotein receptor (ASGPR) [55,56]. Specifically, heparinase I treatment of susceptible hepatoma cell lines significantly reduced VLP binding, as well as HEV infection [57].

Non-enveloped HEV particles are internalized into hepatocytes via clathrin, heparan sulfate, integrin alpha 3, and dynamin 2-dependent endocytosis [34,58,59,60]. In addition, proof indicates that quasi-enveloped HEV penetrates cells through endocytosis and then undergoes membrane degradation inside the endolysosome [33,38]. Notably, both non-enveloped and quasi-enveloped HEV have been demonstrated to bind to heparan sulfate proteoglycans, specifically syndecans, which are present on the outer layers of a variety of cells [34,57] and are known to mediate cell attachment of different types of viruses [54,55,56,57,58]. This engagement causes endocytosis through receptors, which leads to viral host cell internalization. However, the exact mechanism of HEV capsid uncoating is still unknown.

GRP78 is a molecular chaperone that is located in the endoplasmic reticulum. It is also known as binding immunoglobulin protein (BiP). The existence of GRP78 on the outer layer of cells has been stated and linked to several enveloped and non-enveloped viruses’ binding and entrance [61,62,63,64].

ASGPR is a type of protein receptor that connects glycoproteins lacking sialic acid modifications on the basolateral surface of hepatic cells. Coimmunoprecipitation, pull-down, and ELISA analysis have revealed an intimate relationship between the ASGR1-ASGR2 ectodomains and HEV ORF2 [56]. Ectopic ASGRP expression raised HEV adhering in HeLa cells, whereas ASGRP exhaustion decreased HEV attaching yet did not affect virion discharge in PLC/PRF/5 cells. HEV binding to PLC/PRF/5 cells was also reduced by anti-ASGPR 229 antibody and purified ASGPR ectodomain.

Although ATP synthase is primarily a protein found in mitochondria, a small portion is expressed on the external layer of cells and has been linked to other viral infections [65]. The function of ATP5B in HEV entrance was confirmed using antibody/siRNA-mediated systems and pathogenic HEV derived from the feces of patients with hepatitis E [66].

Recently, ITGA3 (Integrin Alpha 3) was shown to be a HEV entrance component of PLC/PRF/5 cells [67]. Integrins are a type of transmembrane protein found on the cell surface of various tissues (e.g., the gut) that can bind to the extracellular matrix, cell surface, and the actin microfilaments of the cytoskeleton [68,69,70], which are known to be sites of HEV replication and infection [43,71]. A recent microarray study compared gene expression in permissive versus non-permissive cells [67]. ITGA3 excessive expression in non-permissive cells enabled infection solely of non-enveloped HEV. Accordingly, in permissive cells, knocking out the ITGA3 gene hindered non-enveloped HEV yet not eHEV admission [67]. However, ITGA3 expression is low in some organs, such as the liver [72,73,74], suggesting that ITGA3 functions as a co-factor for HEV entrance instead of as the primary receptor.

The Rab5 and Rab7 GTPases, which play a role in endosomal transport and acidification, are necessary for eHEV cellular compartment entrance, implying that the endosome must transition to a more acidic cell compartment to allow the uncoating process to take place [34]. Endosomal trafficking appears to be required for cell entry by HEV virions, which can be decreased by inhibiting endosomal acidification. Indeed, culture cells treated with the bafilomycin A1/NH4Cl (lysosomotropic agents) dramatically reduce eHEV’s ability to infect cells, implying that viral entry needs endosomal acidification. Low pH, on the other hand, is insufficient for eHEV cell access because it does not affect the density or infectivity of eHEV virions [34].

An independent study confirmed that HEV-like fragments travel to Rab5-positive spaces before being degraded in acidic lysosomal compartments [60]. The present investigation discovered that actin, the membrane-derived cholesterol, and the pathway governed by PI3K are required for HEV incorporation and spread of infection, though a low pH environment is not. Notably, lysosomal lipid degradation is required to get into eHEV. The loss of the late endosomal (and lysosomal) Niemann–Pick disease type C1 protein, which plays a role in cholesterol removal, substantially decreased eHEV infection [34]. Furthermore, inhibiting lipid degradation with a lysosomal acid lipase inhibitor decreased eHEV cell entrance in a dose-dependent fashion [34]. Furthermore, treating cells with cholesterol-sequestering agents significantly reduced VLP uptake [60]. Additionally, cells treated with a unique inhibitor of lysosomal acid lipase, an enzyme crucial to the processing of lipids whose activity hydrolyzes cholesteryl esters and triglycerides in lysosomes [75], showed a dose-related decrease in eHEV infectivity. Accordingly, there was no reduction in non-enveloped HEV spread [34].

It was recently discovered that blocking Rab5 and Rab7, as well as lysosomotropic factors, does not affect naked HEV infectivity [34]. These findings imply that naked HEV particles are uncoated before entering the Rab5+ space and that capsid co-localization to Rab5 is most likely due to empty capsids that remain in the endocytic pathway after genome release. As seen in other non-enveloped viruses [76,77,78], the viral capsid could come into contact with a mysterious host element and encounter the structural alterations needed for uncoating and genome release into the cytoplasm [79].

ORF3, an eHEV protein that interacts with the capsid [80], has been shown to interact with ORF2 and may disrupt receptor binding [80]. Also, ORF3 has ion channel activity, which is required for HEV egress [31]. Its contribution to eHEV entrance, uncoating, and genetic discharge is still unidentified.

More recently, the Epidermal Growth Factor receptor (EGFR) was suggested to be connected with the HEV admission phase in HepG2 cells, a low invasive HCC cell line, since downregulation of EGFR by siRNA affected viral entry. On the other hand, EGFR has already been involved in the entry process of hepatitis B virus (HBV) and hepatitis C virus [81,82]. These findings could open further therapeutic approaches using EFGR inhibitors in patients with CHE.

Although these findings are encouraging, further study will be needed to verify the importance of these elements in HEV cell adherence and permission during HEV spread of infection, ideally in primary human hepatic cells.

### 3.3. Virion Assembly and Infectious Particle Release

To ensure successful transmission, viruses have created techniques to take control of and modify host cell pathways.

HEV replicase proteins are encoded by ORF 1. After the viral host cell internalization via endocytosis, the translational system generates the ORF1 polyprotein, which moves the HEV RNA replication process. A negative-strand RNA intermediate is converted into a full-length genomic RNA and a subgenomic RNA type during this phase [80,81].

The biological mechanism beneath the metabolism of HEV polyprotein remains one of the most contentious concerns in HEV biology due to its implication as a potential therapeutic target. A previous study concluded that the expressed ORF1 gene product is not proteolytically processed [83]. Several studies have shown that the ORF1 polyprotein is proteolytically processed in vaccinia virus, baculovirus, and eukaryotic expression systems, but it is unclear whether this proteolysis is caused by viral or host proteases [84].

The latest study endorses the theory that pORF1 is missing HEV protease activity and is processed by host proteases [85]. Accordingly, ORF1 expression in cell-free and prokaryotic systems does not result in polyprotein processing [85]. Research additionally supported the function of host protein proteases that participate in blood the coagulation process, such as cellular serine proteases Xa (in the papain-like cysteine protease domain) and thrombin (in the X domain), in processing the HEV ORF1 polyprotein [85]. To further investigate the polyprotein processing of HEV ORF1, a novel BacMam strategy was recently used, in which the entire HEV genotype 3 genome was cloned into a BacMam vector [85]. Specifically, Huh7 immortal cell lines made up of epithelial-like and tumorigenic cells were infected with a recombinant baculovirus containing the HEV genome, obtaining several protein fragments of 18, 35, 37, and 56 kDa, corresponding to papain-like cysteine protease, methyltransferase, RNA-dependent RNA polymerase, and ORF2, respectively, indicating that proteolytic processing occurred [85].

ORF2 and ORF3 proteins are produced by translating subgenomic RNA. Later stages of the HEV life cycle include viral assembly and the release of newly produced virions.

Virion assembly is the encapsulation of genomic HEV RNA in the capsid. While the exact location of HEV assembly is undetermined, it is probably linked to distinct replication structures. Even though the underlying processes of human HEV assemblage are unresolved, preliminary studies discovered that RNA and the ORF2 protein can assemble themselves into virus-like particles (VLPs) without RNA in insect cell membranes [21,82].

HEV generates two isoforms of the ORF2 capsid protein, which operate in various roles throughout the HEV lifecycle [27]. The infectious ORF2 protein is a structural component of virions, whereas the genome-free secreted and glycosylated ORF2 proteins most likely serve as a humoral immune decoy [27].

ORF2 protein moves through the nucleus early during infection to control specific cellular functions, such as the infected cell’s antiviral responses. There have been identified factors that influence ORF2 nuclear imports and exports [27]. The N-terminal region of ORF2 contains an Arginine-Rich Motif (ARM), which facilitates nuclear import. Furthermore, the ARM plays an important role in fine-tuning the partitioning and stoichiometry of the ORF2 protein across the nuclear, cytosolic, and reticular pathways [27]. Additionally, the signal peptide and ARM work together to control the fate of the ORF2 protein. Indeed, in addition to mediating ORF2 targeting the endoplasmic reticulum membrane, the signal peptide is likely capable of forming a reverse signal-anchor topology. This topology inversion is driven by ARM’s flanking charged residues following the positive-inside rule, resulting in the ORF2i protein being anchored to the cytosolic side of membranes [27].

There are considerable types of the ORF2 protein, one of which plays a role in virion development [86,87,88,89]. A specific shape, which contains a shortened and inactive signal peptide, is currently under discussion if it is a product of alternative start site translation [26] or proteolytic cleavage [87]. The non-glycosylated ORF2 protein is thought to be the major internal shape encountered within the cytosol and cell nucleus [87,88,89].

While the nuclear ORF2 protein’s function is unresolved, the nucleocytoplasmic ORF2 transporting is presumably mediated by the nuclear import and export apparatus. The glycosylated form of ORF2 protein is quickly secreted extracellularly (as opposed to the non-glycosylated form that packages the HEV genome) [26,86,87,90,91]. This entails the translocon recognizing its signal peptide, translocating into the endoplasmic reticulum lumen, cleavage by signal peptidase, sialylation, and Nand O-glycosylation by still unknown glycosyl-transferase enzymes in the endoplasmic reticulum and Golgi apparatus [87], and secretion.

The ORF2 protein is glycosylated in no fewer than two distinct states, the smallest of which could be the result of division by furin-like enzymes at the RRR pattern [88], meaning that the ORF2 protein may play multiple roles in genome packaging mediated by various host variables.

Many studies found that ORF3 is required for viral particle egress and biogenesis of lipid membrane-wrapped HEV particles, which are now known as quasi-enveloped particles.

Because genes with a mutated ORF3 start codon produce infectious components, HEV assembly solely necessitates the non-glycosylated ORF2 material and the viral RNA. These, however, are not released by the cell [92,93]. The infectious form of HEV is thought to be discharged from the basolateral as well as apical sides of the liver cells, located adjacent to the liver sinusoids and bile canaliculi, respectively [43,94,95].

The current model of eHEV biogenesis entails viral hijacking of the cellular endosomal sorting complex required for transport (ESCRT) apparatus (Figure 2), resulting in the formation of an exosome-like vesicle comprising the viral capsid and ORF3 protein [96].

The ESCRT machinery is made up of four structures that take part in membrane remodeling, ESCRT-0 through ESCRT-III [97]. These complexes can also recruit accessory proteins, such as Alix and Vps4, which interact with the ESCRT-III complex [98]. Hrs, an ESCRT-0 member, and Vps4 were also discovered to be needed for HEV particle output [37,99].

Furthermore, the envelope surrounding the virion is derived from exosomes, which are small vesicles produced by the ESCRT machinery from multivesicular bodies (MVB) [37,99].

To combine with the outer layer of the plasma membrane and emit its components into the extracellular environment, these MVBs necessitate the Rab27a proteins, which were demonstrated to interact alongside the ORF3 protein [37,99]. A PSAP late domain pattern at the C-terminal of the ORF3 protein primarily connects with the cellular TSG101 protein, an ESCRT-I protein, which then attracts ESCRT-II and -III structures to stimulate HEV capsid developing into MVB [100]. Following MVB fusion with the plasma membrane, single membrane-encased eHEV particles are released into the circulating blood or into the bile duct, where bile acid destroys the quasi-envelope [35,37].

As a result, the high viral load of non-enveloped HEV virions observed in feces is most likely due to the destroying process of the quasi-envelope shell of eHEV particles by bile acid, which is primarily expelled via the bile canaliculi from hepatocytes across the apical membrane [101]. Hence, HEV is shed in the feces of infected individuals as stable, non-enveloped virions with their genome encapsulated in a naked protein shell [101]. Likewise, because of the detergent action of bile, naked virions are far more resilient in the surrounding environment, ensuring a direct and easier host spreading.

Additionally, the viral protein ORF3 is required for HEV infectious virions to be secreted, possibly by connecting the capsid to host egress factors. Furthermore, ORF3 protein serine phosphorylation (by p34cdc2 and mitogen-activated protein kinases) may result in an interaction with non-glycosylated ORF2 protein [80,102].

In addition, the ORF3 protein recruits Tsg101, a member of the endosomal organizing complex required for ESCRT-I, via a highly preserved PSAP motif [99,100,103]. The presence of this pattern in the C-terminus of the ORF3 protein is crucial for HEV discharge [100]. Similarly, other viruses with envelopes (such as HIV-1 or Ebola) require distinct proline-rich patterns (or “late domains”) for budding [103,104].

According to existing proof, none of the HEV proteins are present on the eHEV outer membrane, but ORF3 protein has been recognized beneath the quasi-enveloped type [95,105,106]. Palmitoylation of the ORF3 protein’s N-terminal cysteine residues mediates membrane association and stability, as well as virus secretion [107]. As a result, a membrane topology with ORF3 protein completely on the cytosolic side that corresponds to the exosome lumen was provided [107], and this is confirmed by the PSAP motif’s relationship to Tsg101. As a result, a membrane topology in which ORF3 protein is entirely on the cytosolic side, corresponding to the exosome lumen, has been proposed [107], which is supported by the interaction of its PSAP motif with Tsg101. S-palmitoyl-transferase-mediated palmitoylation is a reversible change process to a protein that takes place in the cytosol and raises a protein’s hydrophobic nature while also contributing to membrane association [108].

Host proteins, especially exosomal proteins, can be observed on eHEV due to their origin. Of course, viral fragments provided from ORF2/ORF3 protein-positive infected cells have been identified as having unique exosomal elements (i.e., tetraspanins, CD63, CD81, and CD9, as well as Tsg101 and Alix) [37,95,109]. Exosomes containing HEV genetic material have been displayed to spread other viruses productively and to regulate cell reactions [110].

Given that HEV may benefit from the quasi-enveloped nature of bloodstream virions, including immunity from neutralizing antibodies, their production may serve some function other than propagating HEV throughout the liver.

On the other hand, a fascinating experimental study shed light on a potential mechanism of HEV-associated neuroinvasion [3]. The study found that quasi-enveloped and naked HEVs can cross the blood–brain barrier without a TNF-α mediated process and infect brain microvascular endothelial cells in vitro [3].

### 3.4. Therapeutic Options for HEV Infection

The HEV’s multifaceted infectiousness and the lack of a cell culture system have limited the attempts to adequately dissect the interaction with the host and to set up antiviral strategies, particularly regarding the inhibition of viral entry. However, recent innovations in cell cultures showed that HEV has an array of replication phases similar to those found in other RNA viruses. Consequently, although substances inhibiting viral entry are not yet at hand for clinical use, several antivirals already used with other RNA viruses and other still experimental drugs in the preclinical phase could offer a highly desirable therapeutic option, as chronic HEV infection with genotype 3 has been linked with chronic liver disease and cirrhosis development in immunocompromised patients [111].

Early experimental data with sofosbuvir [112] were not confirmed in clinical studies [113]. Sofosbuvir monotherapy in 10 immunocompromised patients obtained only a moderate decrease in viremia with a rebound after treatment completion [113].

Non-pregnant patients with chronic HEV infection were shown to benefit from a 12-week course of ribavirin (RBV) monotherapy [111]. RBV is a guanosine nucleoside analog that acts as a viral polymerase inhibitor, and it has been usually given to solid organ transplant recipients in conjunction with immunosuppressive therapy reduction [114,115,116,117,118,119,120,121,122,123,124,125,126,127,128,129]. Table 1 contains details about the described drugs and their mechanisms of action in immunocompromised patients.

Although the mechanism of action is not well defined [130], Marion et al. recently showed that RBV inhibited HEV secretion from the basal side of intestinal cells by more than 80% but only by 20% from the apical side. While this disparity remains unexplained, it highlights the importance of future studies evaluating the efficacy of antiviral drugs in both the liver and the gut [43]. Interestingly, the same authors showed that the HEV in the intestinal reservoir might be partially resistant to ribavirin, thus offering a possible explanation for the endogenous reinfections ensuing ribavirin withdrawal in chronically HEV-infected individuals [43]. Of note, the emergence of viral mutations may contribute to resistance against ribavirin treatment.

Combination treatments with ibrutinib and RBV revealed that ibrutinib had no effect on RBV’s antiviral effect and did not directly promote HEV replication in an elderly patient with lymphoplasmacytic lymphoma [127].

In terms of other potential substances targeting non-entry viral sites, 3-(4-Hydroxyphenyl) propionic acid, a HEV methyltransferase inhibitor, revealed a substantial virological response in an in silico analysis. This substance, however, will require additional validation by in vivo testing in animal models and pharmacokinetic/pharmacodynamic investigations [128].

A recent study [129] showed that the antimalarial Artesunate was able to inhibit HEV replication by directly blocking both the helicase and the RNA-dependent RNA polymerase. A further element of interest about this drug is represented by the fact that it is safe in pregnant women, who are well-known high-risk individuals. However, it may be moderately mutagenic, tumorigenic, and hepatotoxic if utilized in elevated dosages for a lengthy period, thus requiring further testing in animal models.

Because HBV and HEV endemicity overlap significantly in many Asian countries where double HEV-HBV infections can occur, HEV superinfection in patients with chronic hepatitis B can result in acute exacerbation of underlying viral hepatitis, raising the chance of developing decompensated cirrhosis and mortality while displaying a subclinical course of acute HEV infection during pregnancy [131].

To reduce the likelihood of HEV epidemics, preventive measures should be carried out, with investigational HEV vaccines playing a critical role. HEV vaccination has been developed and is being used in China, but its efficacy in CHB patients has yet to be established in the United States [131].

Several HEV vaccines are currently in development, with some already available and demonstrating promising results. A 2007 phase-II trial, despite a high vaccine efficacy, has previously failed due to a lack of commercial value of HEV recombinant protein [132]. In 2010, a vaccine obtained from the ORF2 capsid protein of HEV genotype 1 was developed and licensed in China under the name Hecolin^®^ [133]. All HEV vaccine trials showed cross-genotype activity because HEV genotype 4 is the most common in China [134]. After nearly five years of vaccination, this vaccine provided long-term HEV protection in most previously seronegative patients [135]. Additionally, it was predicted that anti-HEV immunogenicity could be maintained for up to 30 years following vaccination [136]. Notably, many HEV vaccine studies have been conducted in large Asian populations, demonstrating high efficacy and safety even in preventing maternal and neonatal deaths in non-pregnant women [137] (NCT03168412, NCT02417597, and NCT02759991). Regardless, a phase Ia/Ib trial in the United States with 25 healthy non-pregnant females confirmed a total immunoglobulin serological response to the Hecolin^®^ vaccine (NCT03827395). Two further vaccine candidates are being tested in clinical trials with VLPs from insect cells and *E. coli* [138].

## 4. Conclusions and Future Directions

The considerable and changing epidemiologic scenario and the threat of HEV transmission highlight the significance of future HEV prophylactic healthcare measures.

The evidence indicates that HEV can infect intestinal cells, supporting the idea that the gut is the site of initial infection and viral amplification before being transported to the liver [71]. As a result, the gut may act as a reservoir for HEV replication, especially during chronic infection, which, together with the emergence of viral mutations, could explain viral relapses in some treated patients.

Specifically, the cell access strategies utilized by non-enveloped and quasi-enveloped HEV components diverge, and HEV entrance necessitates lysosomal degradation of the viral membrane. Of course, understanding the entry mechanisms of both HEV virion types will depend on discovering their specific cell-surface receptors.

Even though HEV investigation is a quickly expanding domain, the current knowledge of the virus’s life cycle must be enhanced. We are missing critical information on how HEV viruses enter cells, such as cellular receptors and viral RNA uncoating. Future research should also focus on the biological processes and distinct compartments within hepatic cells that handle the replication of RNA and viral assemblage.

While HEV release constitutes one of the most extensively researched phases in the virus’s life cycle, several aspects, particularly the contribution of host-related variables and the nature and function of quasi-enveloped particles, persist unidentified, limiting the discovery of HEV-specific antiviral agents. Given HEV’s skill to reproduce in a variety of tissues and infect an extensive number of beings, it might hypothesized that HEV host dependency may not be highly selective, enabling the virus to cross biodiversity barriers.

Nowadays, research studies on the HEV biological clock rely on laboratory system models that suffer from limitations, particularly in terms of host factors present in differentiated hepatocytes. For example, cyclophilin inhibitors were found to have a stimulatory effect on HEV replication in hepatoma cells [71]. This was demonstrated in stem cell-derived hepatocyte-like cells using a modified in-lab cell clone infectious model but not with authentic HEV isolates [139].

Future enhancements to in vitro experiments ought to encompass the application of naturally produced HEV isolates together with original and hepatocyte-like cells derived from stem cells, polarized cell cultivation models, and both ex vivo/in vivo infection experiments to verify the laboratory outcomes. Key findings in hepatic samples from hepatitis E individuals must eventually be confirmed.

To acquire a more comprehensive and uninfluenced picture of the host-related variables engaged in the HEV life cycle, cutting-edge methods that incorporate CRISPR/Cas9-based genome-wide examination (that have advanced our understanding of virus entrance [140,141], reproduction [142,143], and the designation of unique therapeutic targets [144] and proteomic proximity determining methods (as successfully employed for characterizing the microenvironment of coronavirus replication complexes [145])) are expected to be used.

In the future, incorporating enhanced HEV simulators and technological innovations should produce a greater comprehension of the host-related variables necessary for effective HEV infection.

Ultimately, subsequent studies emphasizing the immune-mediated effects of HEV infection will probably aid in developing personalized therapies, enhancing the clinical outcomes for individuals with acute and chronic HEV hepatitis and conceivably preventing HEV-associated liver cancer.

## Figures and Tables

**Table 1:** Evidence for HEV drug inhibitors with their mechanism of action in immunocompromised patients.

Author	Year	Study Type	Immunosuppressed Patient Group	N. of pts	HEV Genotype (N. pts)	Drug	Mechanism of Action	Dosage/Daily	Treatment Duration (mo)	Virological Response, %	Adverse Events
Kamar [114]	2010	Pilot study	Kidney transplantation/simultaneous kidney-pancreas transplantation	6	3f (4), 3 c (2)	RBV monotherapy	Guanosine nucleoside analog (viral polymerase inhibitor)	600–1000 mg	3	100	Anemia
Mallet [115]	2010	Case report	Simultaneous kidney-pancreas transplantation, hematological disease	2	3f (1), 3c (1)	RBV monotherapy	Guanosine nucleoside analog (viral polymerase inhibitor)	600–1000 mg	3	100	Not relevant
Alric [116]	2011	Case report	Hematological disease	1	3c	RBV monotherapy	Guanosine nucleoside analog (viral polymerase inhibitor)	600–1000 mg	3	100	Not relevant
Chaillon [117]	2011	Case report	Heart transplantation	1	3c	RBV monotherapy	Guanosine nucleoside analog (viral polymerase inhibitor)	600–1000 mg	3	100	Well-tolerated anemia
de Niet [118]	2012	Case report	Kidney transplantation	1	3	RBV monotherapy	Guanosine nucleoside analog (viral polymerase inhibitor)	600–1000 mg	3	100	Not relevant
Del Bello [119]	2012	Case report	Liver transplantation	1	3f	RBV monotherapy	Guanosine nucleoside analog (viral polymerase inhibitor)	600–1000 mg	3	100	Not relevant
Pischke [120]	2012	Prospective	Heart transplantation	4	n.a.	RBV monotherapy	Guanosine nucleoside analog (viral polymerase inhibitor)	600–1000 mg	5	75	Not relevant
Hajji [121]	2013	Case report	HIV	1	3f	RBV monotherapy	Guanosine nucleoside analog (viral polymerase inhibitor)	600–1000 mg	3	100	Not relevant
Junge [122]	2013	Case report	Liver transplantation	1	n.a.	RBV monotherapy	Guanosine nucleoside analog (viral polymerase inhibitor)	600–1000 mg	6	100	Not relevant
Koning [123]	2013	Retrospective	Heart transplantation	4	3	RBV monotherapy	Guanosine nucleoside analog (viral polymerase inhibitor)	600–1000 mg	3–12	75	Not relevant
Neukam [124]	2013	Case report	HIV	1	3	RBV monotherapy	Guanosine nucleoside analog (viral polymerase inhibitor)	600–1000 mg	6	100	Not relevant
Pischke [125]	2013	Prospective case series	kidney transplantation, heart transplantation, lung transplantation	11	n.a.	RBV monotherapy	Guanosine nucleoside analog (viral polymerase inhibitor)	600–1000 mg	5	82	Not relevant
Riezebos-Brilman [126]	2013	Case report	Lung transplantation	2	3	RBV monotherapy	Guanosine nucleoside analog (viral polymerase inhibitor)	600–1000 mg	4	100	Not relevant
Schlevogt B [127]	2019	Case report + in vitro studies (hepatoma cell line HepG2)	Lymphoplasmacytic lymphoma	1	3c	RBV	Guanosine nucleoside analog (viral polymerase inhibitor)	600–1000 mg	2	100	Severe exanthema
					1–3	Ibrutinib	BTK inhibitor	3.33 microM	48 h	Moderate antiviral effect against HEV replicating isolates	No cytotoxicity
Hooda P [128]	2022	In silico (Docking analysis)	n.a.	n.a.	N.A.	HPPA	HEV MTase inhibitor	800 M	n.a.	HEV-RNA copies decreased significantly from ~3.2 × 106 in untreated cells to ~4.3 × 102.8 copies	Because the homology between human and HEV-MTase is so low, HPPA is unlikely to affect the host Mtase
Bhise N [129]	2023	Drug repurposing strategy: experimental + in silico (Docking analysis)	n.a.	n.a.	1–3	ART	(A) binding to the HEV helicase active site, potentially affecting ATP hydrolysis activity; (B) inhibition of the HEV RdRp	19.5 μM (EC50) and 78 μM	n.a.	(A) ART showed a 24% and 55% inhibition of the helicase; (B) ART showed 26% and 40% inhibition of the RdRp activity	In silico toxicity studies revealed no risk of reproductive or developmental toxicity

HEV, Hepatitis E virus; RBV, ribavirin; HIV, human immunodeficiency virus; BTK, Bruton’s tyrosine kinase; HPPA, 3-(4-Hydroxyphenyl)propionic acid; Mtase, methyltransferase; ART, Artesunate; RdRp, RNA-dependent RNA polymerase, n.a. = Not available.
